# History of diabetes is associated with reduced likelihood of achieving PASI75 and PASI90 at 12 months among psoriasis patients treated with biologics: A prospective analysis in the CorEvitas Psoriasis Registry

**DOI:** 10.1016/j.jdin.2023.07.017

**Published:** 2023-08-25

**Authors:** Clinton W. Enos, Yolanda Munoz Maldonado, Hyung-Joo Kang, Robert R. McLean, Abby S. Van Voorhees

**Affiliations:** a Eastern Virginia Medical School Department of Dermatology; b CorEvitas, LLC

**Keywords:** biologics, biologic therapy, diabetes, psoriasis, psoriatic disease, outcomes, treatment response

*To the Editor:* Our previous research in the CorEvitas Psoriasis Registry indicated that among patients with psoriasis who initiated biologic therapy, those with a history of diabetes were less likely to achieve 75% and 90% improvement in psoriasis affected area and severity index (PASI75 and PASI90) by 6 months, respectively.^1^ Analyses stratified by biologic class found that this association was the most apparent within the cohort of patients who initiated interleukin-17 inhibitor (IL-17i) therapy.^1^ The association of diabetes with longer-term biologic treatment response is not known. Further, understanding whether the association between diabetes and treatment response varies among drug classes could help guide treatment choice to optimize management of psoriasis in patients with diabetes. Here, we build on our previous work using longer-term data from the CorEvitas Psoriasis Registry to assess PASI75 and PASI90 after 12 months of biologic treatment in patients with and without diabetes.^2^ We further investigated for the influence of a potential interaction between diabetes and biologic class.

Patients included in the study were those who initiated IL-17i, IL-12/23i, IL-23i, or tumor necrosis factor-inhibitor (TNFi) therapy and completed a 12-month follow-up (9- to 15-month window) between April 2015 and April 2022. Patients were classified by history of diabetes (yes/no). To assess the overall association of diabetes with PASI75 and PASI90 at 12 months, modified Poisson regression was used to calculate multivariable-adjusted relative risks (RR) with 95% CIs. Subsequent modified Poisson models tested for statistically significant interactions (*P* < .05) between biologic class and diabetes.

Of the 17,013 patients enrolled, 4,875 biologic initiators were included (Supplementary Tables I and II, available via Mendeley at https://data.mendeley.com/datasets/pnchbrkxdz/1). At the start of the study, 789 (16.2%) were found to have a history of diabetes, and these patients had higher mean (SD) age (57 [12] vs 50 [14] years), more frequently reported PASI > 10 (35.5% vs 27.7%), and were exposed to ≥2 biologics (48.3% vs 38.4%) compared with those without diabetes.

At 12 months, a smaller proportion of psoriasis patients with history of diabetes achieved PASI75 (53.0% vs 62.3%) and PASI90 (38.4% vs 48.3%) compared to those without. In multivariable analyses, diabetes was independently associated with reduced likelihoods of PASI75 (RR 0.89, 95% CI: 0.80-0.98) and PASI90 (RR 0.83, 95% CI: 0.72-0.95) (Table I) in the overall cohort. In models examining the interaction of diabetes and biologic class, RRs suggested lower likelihood of achieving PASI75 and PASI90 among patients with diabetes who initiated IL-17i, IL-12/23i, and IL-23i classes, although all CIs included the null value (Table II). There was no statistically significant interaction between diabetes and biologic class for either PASI75 (*P* = .59) or PASI90 (*P* = .24) at 12 months (Table II).

**Table I:** Unadjusted and adjusted∗ relative risks (95% CI) for outcome achievement at 12 months following biologic initiation for plaque psoriasis patients with vs without history of diabetes

Outcome	No. of achieved outcomes at 12-mo		Unadjusted RR (95% CI)	Adjusted RR (95% CI)
	Diabetes	No diabetes		
	(*N* = 789)	(*N* = 4,083)		
PASI90, *n* (%)	303 (38.4%)	1,970 (48.3%)	0.80 (0.71-0.89)	0.83 (0.72-0.95)
PASI75, *n* (%)	418 (53.0%)	2,542 (62.3%)	0.85 (0.78-0.93)	0.89 (0.80-0.98)

∗ Adjusted models included the following: Age, sex, race, ethnicity, insurance (this was a variable created so that the levels were mutually exclusive, and they included Private only, Medicare only, Medicaid only, none, and 2 or more insurances), education, work status, geographic region, smoking status, drinking habits, obesity, psoriasis duration, comorbid psoriatic arthritis, biologic experience, and drug class.

**Table II:** Relative risks (95% CI) for outcome achievement at 12 months following biologic initiation for plaque psoriasis patients with vs without history of diabetes, stratified by biologic class∗∗

Outcome	No. of achieved outcomes at 12-mo		Adjusted RR (95% CI)	*P* value for interaction
	Diabetes	No diabetes		
	(*N* = 789)	(*N* = 4,083)		
PASI90, *n* (%)				.238
TNFi	43 (37.7%)	229 (39.3%)	0.99 (0.78-1.26)	
IL-17i	126 (37.5%)	757 (46.9%)	0.81 (0.62-1.06)	
IL-12/23i	15 (30.0%)	178 (46.9%)	0.65 (0.38-1.09)	
IL-23i	119 (41.2%)	806 (53.6%)	0.84 (0.61-1.15)	
PASI75, *n* (%)				.586
TNFi	63 (55.3%)	329 (56.2%)	0.99 (0.75-1.30)	
IL-17i	176 (52.4%)	975 (60.4%)	0.90 (0.72-1.12)	
IL-12/23i	22 (44.0%)	239 (62.9%)	0.78 (0.53-1.16)	
IL-23i	157 (54.3%)	999 (66.4%)	0.88 (0.67-1.16)	

Covariates included: Age, sex, race, ethnicity, insurance (this was a variable created so that the levels were mutually exclusive, and they included Private only, Medicare only, Medicaid only, none, and 2 or more insurances), education, work status, geographic region, smoking status, drinking habits, obesity, psoriasis duration, comorbid psoriatic arthritis, biologic experience, and drug class.

∗∗ Estimates derived from single regression model with diabetes X biologic class interaction terms and covariates.

We provide evidence for an independent association between diabetes and poorer long-term response to biologic therapy among patients with psoriasis at 12 months. The mechanism behind this association is not currently known. Importantly, we found no evidence indicating that this association varies among biologic classes. Potential limitations of this study include modest sample sizes of individual drug classes, reduced generalizability due to a nonrepresentative sample and exclusion of patients with missing visits, and inability to specify type 1 or 2 diabetes. Additional research is needed to better understand the relationship between diabetes and treatment outcomes to provide optimal care for all patients with psoriasis.

## Conflicts of interest

Abby S. Van Voorhees declares (Grant/Research Support) Celgene, Lilly, AbbVie; Consultant: Amgen, Boehringer Ingelheim, BMS, UCB.

Clinton W. Enos declares (Investigator/advisory board) Amgen. (Spouse receives stock) Realta LifeSciences, Yolanda Munoz Maldonado, Hyung-Joo Kang, and Robert R. McLean are CorEvitas, LLC employee.
